# Changes in Home and Health over Nine Years among very Old People in Latvia – Results from the ENABLE-AGE Project

**DOI:** 10.1007/s10823-016-9311-3

**Published:** 2016-12-27

**Authors:** Charlotte Löfqvist, Signe Tomsone, Susanne Iwarsson, Vibeke Horstmann, Maria Haak

**Affiliations:** 1 Department of Health Sciences, Faculty of Medicine, Lund University, PO Box 157, SE-221 20 Lund, Sweden; 2 Department of Rehabilitation, Riga Stradins University, Dzirciema 16, Riga, LV-1007 Latvia

**Keywords:** Age/ageing, Demography, Health planning, Health services, Public health

## Abstract

To meet the needs of an increasing, heterogeneous, ageing population it is imperative to understand links between home and health. In Latvia, only limited research targeting the health and home situation of very old people is available. Consequently, the aim of this study was to describe how the home environment and aspects of health have changed over nine years between 2002 and 2011 for very old people in Latvia, living in their home environment. This study is based on the Latvian part of the cross-national European ENABLE-AGE Project comprising data on objective, as well as perceived, aspects of home and health. Longitudinal data from those involved on both data collection occasions (*N* = 59) was used. At the nine-year follow-up, participants were between 86 and 90 years of age, still living in their own homes. The results show that not only health aspects varied along the ageing process, objective and perceived aspects of home also changed. The physical as well as the cognitive and emotional bonding to the home significantly increased i.e. aspects of meaning such as familiarity and feeling safe in your home, privacy and independence became more important for the very old participants over time. Life satisfaction increased over the years even though objective health factors decreased. Since aspects of home as well as health can be assumed to impact on the outcome of ageing, the situation for this age group in Latvia must be further studied in order to develop suitable and appropriate social and health services, policies and living conditions.

## Introduction

Within the Latvian community, demographic changes along with greater life expectation has placed pressure on health and social services in order to meet the needs of an increasing, heterogeneous, ageing population. In Latvia, the population who are 80 years and older will grow dramatically over the next few decades to 13% by 2035, and will comprise 20% of the working age population in 2050 (European Commission 2015). As in other European countries, the proportion of those ageing in their own private homes is increasing, and the need to expand the knowledge base on the life conditions for the very old is imperative in order to support active, healthy ageing. Since Latvia is one of the prioritised states within European Union (EU) under political and economic transformation and limited research targeting very old people’s health and home situation is available, this is an important area to explore further. One important goal in improving health is to create environments supporting healthy living and subjective well-being. According to the EU, participation and inclusion among the ageing population are particularly important aspects (European Commission 2013, 2016 ). Also, since old people may have suffered from changes in e.g. living conditions along with the Eastern European transition to a greater extent than other groups, this makes it an important field to study (Helasoja et al. 2006).

Studying changes in home and health over time highlights the importance of contextual understanding of laws and regulations as well as other societal development, since they are closely linked (Clarke and Nieuwenhuijsen 2009). Latvia is located on the eastern Baltic coast and has faced a complex history during the 1900s. During the century Latvia has been occupied by several countries before regaining their independence again in 1991 and the political and economic situation in Latvia were changed into a parliamentary republic governed by the State President and parliament. Since 1991, society has been engaged in transforming its health care and social services system towards a centralization of financial resources and a western European social insurance funding model (Aidukaite 2003; Eurpean Commission 1997). The government has created and adopted several new social security laws on e.g. state pensions, social tax and social and medical protection of disabled people. Later, in 2002, a law *On Social Services and Social Assistance* was implemented, enabling old people to receive support and care in their homes, have access to activity (day) centres, meals on wheels and a retirement pension and technical assistance (Hantrais 2002). Moreover, prevention strategies to emphasise public health over the course of life and improvements to the health care system were developed. Since then, the financial crisis in 2007 has reduced the welfare level and the situation has deteriorated rapidly (European Commission 2016). The cost for health and social care in 2008 comprised in total 12.5% of GDP (the health care part 4%), in comparison with all EU countries, where it comprises 25% of GDP (health care 7.5%). In Latvia, the development of the primary health care system and access to general practitioners (GP) and social services have been prioritised issues, resulting in a high level of social change and policy development (Bankauskaite and O’Connor 2008). However, change has led to increased inequality e.g. patient fees for medical care and treatment has increased in recent years. Moreover, there are differences in service provision between regions in the Latvian context and private healthcare facilities are also available, providing health services to those who can afford private, out-of-pocket payments (European Commission 2016).

Housing reforms in Latvia since 1992 were marked by an emphasis on privatisation of state and municipal housing. The process resulted in a shortage of affordable housing in urban areas, the deterioration of existing housing of all tenure types and lack of adequate investment mechanisms to sustain the quality and vitality of the housing sector (Eurpean Commission 1997). There is little up-dated information on housing standards in Latvia, however according to the data from the population census of 2000, the vast majority lived in flats while close to 30% of the total population occupied a private family house or part of such. One of the few research projects studying home and health in very old age was the ENABLE-AGE Project conducted in five European countries, Latvia being one (Iwarsson et al. 2007). Numerous studies have been published based on this data describing national differences and similarities (Iwarsson et al. 2016) and one of the most recent showed that participants in the Eastern European samples (Latvia and Hungary) reported lower scores as concerns life satisfaction and higher scores for depression as compared with participants in the Western European samples (Sweden, Germany, and the UK) (Tomsone et al. 2013). Moreover, in Latvia the participants perceived that their control in the home situation was to a greater extent dependent on factors that were beyond their control. Latvian participants were more independent in activities in daily living such as shopping, transportation and cooking, compared with all the other participants. Studying the association between life satisfaction and e.g. living situation and health among Swedish and Latvian women revealed that standard of living was one of the most important factors, along with coping strategies, that were associated with life satisfaction among very old women in Latvia. This is contrast to the Swedish results showing the opposite situation (Horstmann et al. 2012). Studies from the ENABLE-AGE Project have also shown that there are differences in the access and use of assistive technology, Latvia having one of the lowest use rates (Löfqvis et al. 2005). This is in line with other studies exploring variations in late life health among old Europeans where, for example, Ploubidis el al found that 24% of the overall variation in later life health in Europe appears to be due to county level differences such as lifestyle and income (Ploubidis et al. 2012). In general, results are revealing poorer health outcomes for older people in Eastern countries. Even though health has, to some extent, improved in EU countries (Marmot et al. 2012). Ongoing rapid changes in countries in transformation such as Latvia are therefore of particular interest to study from a longitudinal perspective since the transactional and dynamic relationships between a person and his/her environment are well known to impact health in everyday life (Iwarsson et al. 2016). Health outcomes are influenced by many aspects, living standard and living situation are known to be some of them and overall health dynamics has changed worldwide over the latest few decades. For these purposes, both aspects (home environment and health) are crucial to address when targeting health promotion for very old people. The relationship between home aspects and health in very old age has been studied, revealing that those living in more accessible homes, who perceived themselves as meaningful and useful, had a better sense of well-being and independence in everyday life (Oswald et al. 2007b). Overall, the relationship between the person and the physical and social environment becomes increasingly important in later life (Oswald et al. 2007a, b) and the home environment and neighbourhood are important arenas for old people, and may affect participation in everyday life and independence, as confirmed by several studies in Sweden (Haak et al. 2007; Häggblom-Kronlöf et al. 2007).

There is knowledge and experience available worldwide on health dynamics, but specific knowledge on Latvia can provide an evidence base for the development of social policy as well as health and social care practice in the local context. Consequently, the aim of this study is to describe how the home environment and aspects of health have changed over nine years between 2002 and 2011 for very old people in Latvia, living in their home environment.

## Material and Methods

### Project Context and Sample

This study is based on the Latvian part of the cross-national, interdisciplinary European project, ENABLE-AGE; Enabling autonomy, participation and well-being in old age: the home environment as a determinant for healthy ageing (Iwarsson et al. 2005), funded by the European Commission (EC). From start in 2002, five countries (Sweden, Germany, UK, Hungary and Latvia) were involved (*N* = 1918). The longitudinal ENABLE-AGE Project performed several data collections over the years (between two and four follow-ups depending on country involved), but for this specific study we used data from baseline and follow-up nine years later in Latvia. The project as such is described in detail elsewhere (Iwarsson et al. 2005; Iwarsson et al. 2007). One part of the ENABLE-AGE Project comprised a comprehensive longitudinal survey. At baseline, 2002–2003, *N* = 303 were interviewed and nine years later a follow-up was completed. All instruments and questionnaires were available in Latvian as well as Russian, and the language most appropriate for each participant was used for the data collection. This study uses longitudinal data from those involved on both data collection occasions (*N* = 59). For sample description see Table 1.

**Table 1:** Sample description at baseline (*n* = 59)

Characteristics at baseline	Participants
Age (Md,q1,q3)	78 (77–81)
Male, n (%)	6 (10)
Marital status, n (%)	
Widowed	35 (59)
Never married	15 (25)
Divorced or other (e.g. partner living elsewhere)	9 (16)
Satisfaction with income (Md, q1,q3)^a^	2 (0,4)
Type of housing, n (%)	
Multi dwelling	51 (86)
One family house	1 (2)
Other type	7 (12)
No of rooms, Md (q1,q3)	1 (1,2)
Receiving home care and social care, n (%)	18 (31)
Receiving medical care services, n (%)	17 (29)

^a^Scale 1–10, 10 = very satisfied - 1 = very unsatisfied

### ENABLE-AGE Sampling and Data Collection Procedure

The Latvian ENABLE-AGE Survey Study sample was recruited from people aged 75–84 years at baseline, in single-living private households in the urban districts of Riga and Jurmala. Because of the explorative nature of the ENABLE-AGE Project, one important characteristic of the sampling strategy was that it did not intend to be nationally representative. The sampling strategy for the target population was made through public organisations, pensioners’ unions and social services. Formal ethical approval was granted by the authorities concerned.

Data were collected at home visits by a specially-trained interview team consisting of occupational therapists (Iwarsson et al. 2005) on both data collection occasions. In order to reach the participants who were still available for the nine-year follow-up, an information letter was sent out to the study participants who had given their consent to be contacted for additional data collection. When possible, the letter was followed by a phone call or a home visit. If the person agreed to participate, the date and time for the interview were set.

Based on the verification of informed consents from this second ENABLE-AGE Survey Study in 2004, the total number of theoretically-potential participants for the nine-year follow-up was *n* = 211. At the time of the nine-year follow-up, 82 individuals were deceased, 44 people no longer fulfilled the originally inclusion criteria (living alone in ordinary housing) or had declined to participate in repeated studies. Of those declining to participate, the most common reason for dropping out was interview too strenuous (*n* = 11). Six (*n* = 6) reported lack of interest, and four (*n* = 4) poor health. One (*n* = 1) mentioned distrust or fear. This left us with 85 remaining potential participants. Twenty-six (*n* = 26) of these were not possible to reach, even after several attempts.

#### Dropouts

Dropout analysis between baseline and the one-year follow-up revealed no significant differences in age, gender, education, income or overall health and life satisfaction. However, those who dropped out during the first year (*n* = 78) had a tendency towards higher depression scores (*p* < 0.05). Drop-out analysis performed at this nine-year follow-up revealed no significant differences in gender or education level. However, those dropping out after nine years (*n* = 244), were those perceiving their health worse in terms of overall health, physical mobility, hearing and sight.

### Instruments

This study utilised a sub-set of data from the ENABLE-AGE Survey Study comprising well-proven, self-report scales and observational formats on home and health, along with project-specific questions (Iwarsson et al. 2005). In addition, general descriptive data variables used in this study reflected objective, as well as perceived, aspects on home and health.

#### Home Environment

##### Objective Aspects of the Home Environment

The presence of environmental barriers in the home, that is the number of physical barriers indoors, at the entrance, and in the nearby outdoor surroundings of the home (e.g. waste bin, mailbox), in total 182 items were measured by means of the Housing Enabler Instrument (Iwarsson and Slaug 2001). The magnitude of housing accessibility problems was calculated i.e. the relationship between the personal and the environmental components, based on predefined severity ratings that quantify the severity of the Person-Environment fit problems predicted to arise in each case. One component of accessibility consists of physical environmental barriers, in this study referred to as environmental barriers.

##### Perceived Aspects of the Home Environment

Perceived aspects of home were assessed by means of four different instruments reflecting aspects of usability, meaning, control and satisfaction with the home environment, arising from the four-domain model (Oswald et al. 2006).

The Usability of My Home Questionnaire (Fange and Iwarsson 1999; Fänge and Iwarsson 2003), assessed the degree to which the person perceived that the physical environment supported the performance of activities in the home, scored from 1 (not at all suitable) to 5 (very suitable). One subscale including four items (α = 0.84) captures usability in terms of activity aspects in the home, to what extent is the home environment suitably designed. The subscale of physical environment aspects included six items (α = 0.84) (Oswald et al. 2006), e.g. how the usability in the home is perceived - higher sum-score indicates better usability.

The Meaning of Home Questionnaire (Oswald et al. 1999) covered home attachment in terms of bonding to the home, related to physical, behavioural, cognitive and emotional aspects of meaning. Participants were instructed to judge to what extent they personally agreed or disagreed with the statements on a scale from 0 (strongly disagree) to 10 (strongly agree). The physical bonding subscale included seven items such as housing conditions and comfortability (α = 0.69). The behavioural bonding included six items on e.g. being able to manage everyday life (α = 0.67). The cognitive/emotional bonding subscale included ten items on for example familiarity, feeling of safety and privacy (α = 0.66) (Oswald et al. 2006). Higher median indicates stronger attachment to the home.

The Housing-Related Control Belief Questionnaire, rated on a 5-grade scale from 1 (not at all) to 5 (very much) to what extent events in the home were dependent on him/herself or upon external control. For example, by powerful other individuals, by chance, or by faith (16 items, α = 0.67) (Oswald et al. 2006). The median of the sum score of the 16 items was used in the analysis; the higher the median, the more external control of the home.

Housing satisfaction was assessed by a single item retrieved from the Housing Options for Older People (HOOP) questionnaire, graded from 1 to 5; higher scores indicate higher levels of satisfaction. Income satisfaction was assessed by means of a 10-grad scale; 10 = very satisfied - 1 = very dissatisfied.

### Health

#### Objective Aspects of Health

The Activity of Daily Living (ADL) Staircase was used, including five personal ADL (P-ADL) items (feeding, transferring, toileting, dressing and bathing) and four instrumental ADL (I-ADL) items (cooking, using transportation, cleaning and shopping). The administration of the ADL Staircase included observation and interview, by means of a 3-grade scale (independent/partly dependent/dependent (Sonn and Asberg 1991). The ADL items rated as possible to carry out independently or self-perceived difficulty in the performance were further assessed (Iwarsson et al. 2005).

Functional capacity was assessed by means of the personal component of the Housing Enabler, consisting of 13 items on functional limitations assessed as being present or not (Iwarsson and Slaug 2001).

#### Perceived Aspects of Health

Number of symptoms of depression were assessed by means of the Geriatric Depression Scale (Hoyl et al. 1999), i.e. a dichotomy yes or no scale including fifteen items.

Perceived functional independence in ADL was assessed by a single item from the Neuropsychological Ageing Inventory, scored from 0 (totally dependent) to 10 (totally independent) (Oswald 2005).

Perceived health was assessed by the single item retrieved from the SF-36 (Sullivan et al. 1993; 1995), scored from 1 (excellent) to 5 (poor).

A single item on life satisfaction was scored from 0 (very dissatisfied) to 10 (very satisfied). Perceived functional mobility was scored on five-grade scale from 1 (excellent) to 5 (poor) (Iwarsson et al. 2005).

### Data Analyses

Descriptive statistics were used to describe changes over time; median and quartiles on changes between baseline and follow-up. Changes on group-level as well as individual changes are presented. Frequencies of individual changes are also presented for illustrative reasons for items assessed on a five-grade ordinal scale (that is for housing satisfaction, perceived health and functional independence and life satisfaction) and for numbers of ADL activities performed without difficulty. Changes in home aspects are based on values from those who lived in the same home over the entire study period. During the study period, three people of the remaining 59 moved to another dwelling and were therefore not included in the analysis of housing. Changes over time were analysed by means of Wilcoxon signed rank test and sign test. *P*-values <0.05 were set as a significance level.

## Ethics

This study followed the Helsinki Declaration. For data collection in Latvia, formal ethical approval was granted by the Ethics Committee at Riga Stradiņš University. Furthermore, professional discussions and agreements were undertaken between the researchers involved in the ENABLE-AGE Survey Study, including production of joint information sheets and consent forms (Iwarsson et al. 2005).

## Results

At the nine-year follow-up, the participants were between 86 and 90 years of age, and were still living in their own homes. In terms of sociodemographic changes, the participants perceived that their income satisfaction had significantly increased over the years.

### Changes over Time Concerning the Home Environment

The median number of rooms in the participant’s homes stayed as one room over the years. Even so, the number of environmental barriers, in particular indoors and at the entrance, significantly increased over time (Table 2). Turning to perceived aspects of the home, a small decrease in housing satisfaction was observed, however not significant; thirty participants reported no change or slightly higher housing satisfaction while 26 reported less. The physical, as well as the cognitive and emotional, bonding to the home significantly increased i.e. aspects of meaning such as familiarity and feeling safe in the home, privacy and independence became more important for the very old participants over time. Changes in how the participants perceived the usability of the environment did not significantly change. That is, the participants felt that the physical environment supported their performance of everyday activities in the home over time.

**Table 2:** Changes in objective and perceived aspects of home and health, at baseline and at nine years-follow-up, among very old Latvian people, *N* = 59

Home, n = 55^a^	Baseline	Follow-up	Changes on individual level	p-values
Objective aspects, md (q1,q3)				
No of environmental barriers				
Indoor (0–100)	31 (26–34)	32 (29–36)	3 (−1,6)	0.002
Outdoor (0–33)	13 (10–15)	13 (12–15)	0 (−1,3)	0.139
Entry (0–49)	10 (8–13)	14 (12–19)	4 (1,6)	< 0.0005
Total (0–182)	55 (49–63)	65 (60–69)	9.5 (3,16)	< 0.0005
Accessibility score	80 (38–111)	150 (73–237)	54 (−6138)	< 0.0005
Perceived aspects, md (q1,q3)				
Housing satisfaction, md (q1,q3)	4 (2–4)	2 (2–4)		0.100 ^b^
(Frequencies)			Unchanged, n = 14	
			Increased, n = 13	
			Decreased, n = 24	
Housing related control beliefs	2.96 (2.67–3.24)	3.07 (2.75–3.31)	0.06 (−0.10,0.41)	0.222
Meaning of home				
Physical bonding	7.14 (6.50–7.92 )	7.71 (6.78–8.64)	0.42 (−0.39,1.39)	0.023
Behavioral bonding	7.83 (7.00–9.17)	7.50 (6.79–8.87)	0.42 (−1.88,1.42)	0.392
Cognitive emotional bonding	7.80 (7.40–8.70 )	9.05 (8.36–9.60)	1.10 (1.55,2.10)	< 0.0005
Usability in my home				
Activity aspects	4.25 (3.50–4.75)	4.00 (3.33–5.00)	0 (−0.81,0.62)	0.457
Physical environmental aspects	3.82 (3.40–4.33)	4.00 (3.19–4.54)	0 (−0.57,0.59)	0.955
Health, N = 59				
Objective aspects, md (q1-q3)				
No of functional limitations, total, n = 15	2 (2–3)	4 (2–4)	1.0 (−1.0,2.0)	0.012
No of functional limitations, physical,n = 13	2 (2–3)	3 (2–4)	0 (−1,1)	0.211
P-ADL, no of items without difficulty	5 (4–5)	3 (2–4)	-1 (−3,-1)	< 0.0005
Frequencies:			Unchanged, n = 9	
			Better, n = 6	
			Worse, n = 44	
I-ADL, no of items without difficulty	3 (2–4)	1 (0–3)	-1 (−2- (−1))	< 0.0005
Frequencies:			Unchanged, n = 7	
			Better, n = 5	
			Worse, n = 47	
Perceived aspects				
Depression score, md (q1-q3)	5 (2–7.75)	4 (2–7)	0.04 (−3,1.57)	0.397
Perceived health, md (q1–3) (Excellent- Poor)	4 (4–4)	4 (4–4)		0.049^b^
(Frequencies)			Unchanged, n = 41	
			Better health, n = 4	
			Worse health, n = 13	
Perceived functional independence	8 (7–10)	6 (5–8)		<0.0005^b^
(Frequencies)			Unchanged, n = 16	
			Increased indep., n = 7	
			Decreased indep., n = 33	
Perceived physical mobility	4.0 (3.0–4.0)	4 (4.0–4.25)		0.003^b^
(Frequencies)			Unchanged, n = 6	
			Worse mobility, n = 45	
			Better mobility, n = 5	
Life satisfaction	5 (5–7)	7 (5–8)		0.009^b^
(Frequencies)			Unchanged, n = 12	
			Better satisfaction, n = 30	
			Worse satisfaction, n = 12	

For home aspects, due to internal dropouts N varies between n = 40 (housing related control believe) and n = 55

For health aspects, due to internal dropouts N varies between n = 46 (depression score for T3) and n = 59

Changes analysed by means of Wilcoxon signed rank test

^a^ Three participants moved to another dwelling and were not included in the analyses of home. One more participant was excluded due to missing data at follow-up

^b^ Changes analysed by means of sign test

### Changes in Health Aspects

Turning to changes in health factors over time, the level of dependence increased for both P and I ADL activities (Table 2). That is, the participants performed a smaller number of ADL activities without difficulty after nine years. In particular, difficulties in managing shopping and transportation increased. Also the number of functional limitations increased, accordingly. Considering perceived aspects of health reflected by the participants, functional independence decreased for the majority, as did physical mobility. In contrast, satisfaction with life increased significantly i.e. thirty individuals scored higher life satisfaction over the study period. In addition, the majority of the participants perceived no changes in overall health and a slightly decrease in number of symptoms of depression were reported (see Table 2).

## Discussion

From this study we can learn that not only health aspects vary along the ageing process, objective and perceived aspects of home also change over time, even when staying in the same home environment and even in advanced age. Over time, it became increasingly important to live in a home that suited your physical needs. Similarly cognitive emotional aspects such as security and familiarity in the home became increasingly important in the Latvian sample. This kind of findings is vital to be aware of since old people who perceive their home as useful, are independent in everyday activities and feel in control of their home situation, have higher levels of well-being and suffer less from depressive symptoms (Oswald et al. 2007a). It also has to be kept in mind that during the life-span of our participants’, major political and economic changes have been made in the Latvian society that could impact on how old Latvians perceive their health and needs.

In our results, life satisfaction increased over the years even though objective health factors, not surprisingly, decreased. Notable is that even so, a high proportion rated their perceived health as unchanged. Possible explanations for this could be living in a societal context in change, many people and their family members are gradually achieving a better situation. The most important change for older people in Latvia after the regained independence was the pension system reform during the 90-ties and higher pension and better social security could contribute to these findings. After the economic crisis in 2007, society has recovered, possible impacting on the level of satisfaction. In a recent study on life satisfaction among older women in Latvia and Sweden, Horstman et al. (2012) found that the better the financial situation and health conditions, the higher the life satisfaction. In addition, the standard of living and coping also played a role for life satisfaction. That is, aspects in the home and objective and perceived health aspects are interrelated, and how these impact on health over time therefore needs to be further explored.

Usability in the home did not change over time. The very old people in this study seemed to keep on doing their home activities. This can be explained in terms of being capable of adapting to the situation, something that presumably have been necessary for very old people in Latvia to do, in line with societal changes. Even if level of health is decreasing, environmental aspects change, the old person seems to adapt to the situation, keeping up habits and routines. The literature describes evidence for the importance of activity for health (Gallagher et al. 2015) these findings are well in line with ageing theories claiming that activity promotes life satisfaction (Johnson and Mutchler 2013). This might be particular true for very old people in Latvia as they have experienced major political and social changes. Surprisingly, the number of environmental barriers increased over time. This might be due to changes and wear in the physical environment, for example settings in the walking surface and poor maintenance of real estate property resulting in an increased number of environmental barriers. On the other hand, renovations and improvement in accordance with legislation and rules may also contribute to less accessibility for very old people; for example when complex entrance door opening-systems are installed to increase security in the building, the opening system becomes demanding in other ways. In Latvia, many apartment buildings were built in the sixties and seventies. Since then these have been poorly maintained and findings concerning increased environmental barriers are important for authorities in countries like Latvia to take into account as regards the distribution of governmental finances and development of regulations for building construction.

When studying a country such as Latvia, a country that has gone through significant reforms and changes in the latest few decades, the need for contextual understanding is imperative in order to interpret the findings. Socio-economic and political factors, such as poverty and political instability, have exerted a negative impact on population health and the development of social policy has been awarded low priority after gaining independence (Aidukaite 2003; European Commission 2016). Already at baseline for the ENABLE-AGE Project in 2002, the Latvian participants reported worse health than in western European countries (Löfqvist et al. 2005). The transformation in Latvia has implied that housing has become a private responsibility for the individual, just as the responsibility for individual welfare at large has. This is important to be aware of when interpreting the findings on changes in home and health for very old people in Latvia.

As many factors influence health, the process of studying health and well-being in late life is complicated and research need to focus on perceived as well as objective aspects. Moreover, it is imperative to understand the links between home and health since aspects of both can be assumed to impact the outcome of ageing. Interviewing very old people can be a challenge (Iwarsson et al. 2004), dealing with lack of strength and also suspicion or mistrust. Interviews during home visits still represent a quite new occurrence in Latvia. Moreover, poor living situations and cases of violence and criminal offences against older people could strengthen negative attitudes to, and anxiety about, participating in research. At least that is true for these urban regions were this study was performed.

For the nine-year follow-up there was an extensive dropout rate on the instruments reflecting perceived aspects of the home and the findings have to be understood in the light of this limitation. Considering the high age of the participants, it can be assumed that the comprehensive questionnaire used for the project turned out to be too strenuous or complex at that time point of age, threatening its validity. Regarding the statistical testing, the same level of significance was used throughout the tests even though sample sizes differed between items. This might have impacted on which items showed a significant change over time and which did not, and consequently has to be considered in the interpretation of the findings.

In conclusion, this study is unique involving data from very old people in a longitudinal perspective. To the best of our knowledge, no other longitudinal studies with participants of this age group exist taking detailed information on home as well as health aspects into account. However, the situation for this age group must be further studied in order to develop suitable and appropriate services and living conditions for very old people in different European contexts.

## References

[CR1] Aidukaite J (2003). From universal system of social policy to particularistic? The case of the Baltic states. Communist and Post-Communist Studies.

[CR2] Bankauskaite V, O’Connor J (2008). Health policy in the Baltic countries since the beginning of the 1990s. Health Policy.

[CR3] Clarke P, Nieuwenhuijsen ER (2009). Environments for healty ageing: a critical review. Maturitas.

[CR4] European Commission. (1997). Agenda 2000 - Commission Opinion on Latvia’s Application for Membership of the European Union. DOC/97/14: European Commission.

[CR5] European Commission. (2013). *Public Health - good health for everybody*. European Union explained series. [Cited November 2013]. European Commission. Available at http://europa.eu/pol/health/index_en.htm

[CR6] European Commission. (2015). *The 2015 Ageing Report Economic and budgetary projections for the 28 EU Member States (2013–2060)*. European Economy 3. [Cited November 2015]. European Commission. Availabe at http://europa.eu/epc/pdf/ageing_report_2015_en.pdf

[CR7] European Commission. (2016). Comission staff working document. *European commission country Report Latvia 2016*. [Cited June 2016]. European Commission. Availabe at http://ec.europa.eu/europe2020/pdf/csr2016/cr2016_latvia_en.pdf

[CR8] Fange A, Iwarsson S (1999). Physical housing environment: development of a self-assessment instrument. Canadian Journal of Occupational Therapy.

[CR9] Fänge A, Iwarsson S (2003). Accessibility and usability in housing: construct validity and implications for research and practice. Disability and Rehabilitation.

[CR10] Gallagher M, Orla T, Muldoon OT, Pettigrew J (2015). An integrative review of social and occupational factors influencing health and wellbeing. Front Psychology Journal.

[CR11] Haak M, Dahlin-Ivanoff S, Fänge A, Sixsmith A, Iwarsson S (2007). Home as the locus and origin for participation: experiences among very old Swedish people. Occupational Therapy Journal of Rehabilitation.

[CR12] Hantrais L (2002). Central and east European states respond to socio-demographic challenges. Social Policy & Society.

[CR13] Helasoja VV, Lahelma E, Prättälä R, Kasmel A, Klimbiene J, Pudule I (2006). The sociodemographic patterning of health in Estonia, Latvia, Lithuania and Finland. European Journal of Public Health.

[CR14] Horstmann V, Haak M, Tomsone S, Iwarsson S, Gräsbeck A (2012). Life satisfaction in older women in Latvia and Sweden - relations to standard of living, aspects of health and coping behaviour. Journal of Cross-Cultural Gerontology.

[CR15] Hoyl MT, Alessi CA, Harker JO, Josephson KR, Pietruszka FM, Koelfgen M, Mervis JR, Fitten LJ, Rubenstein LZ (1999). Development and testing of a five-item version of the geriatric depression scale. Journal of American Geriatric Society.

[CR16] Häggblom-Kronlöf, G., Hultberg, J., Eriksson, B. G., & Sonn, U. (2007). Experiences of daily occupations at 99 years of age. *Scandinavian Journal of Occupational Therapy, 14*, 192–200. 10.1080/11038120601124448 17763201

[CR17] Iwarsson, S., & Slaug, B. (2001). *Housing Enabler: An instrument for assessing and analyzing accessibility problems in housing*. Nävling and Staffanstorp: Veten & Skapen HB & Slaug Data Management.

[CR18] Iwarsson S, Wahl HW, Nygren C (2004). Challenges of cross-national housing research with older persons: lessons from the ENABLE-AGE project. European Journal of Ageing.

[CR19] Iwarsson S, Sixsmith J, Oswald F, Wahl HW, Nygren C, Sixsmith A, Szeman Z, Tomsone S, Wilkinson N, Hurol Y (2005). The ENABLE-AGE project: multi-dimensional methodology for European housing research. Housing research methodologies.

[CR20] Iwarsson S, Wahl HW, Nygren C, Oswald F, Sixsmith A, Sixsmith J, Szeman Z, Tomsone S (2007). Importance of the home environment for healthy aging: conceptual and methodological background of the European ENABLE-AGE project. The Gerontologist.

[CR21] Iwarsson, S., Löfqvist, C., Oswald, F., Wahl, H-W., Slaug, B., Schmidt, S., Tomsone, S., Himmelsbach, I. & Haak, M. (2016). Synthesizing ENABLE-AGE research findings to suggest evidence-based home and health interventions. *Journal of Housing for the elderly,* in press doi:10.1080/02763893.2016.1198742

[CR22] Johnson KJ, Mutchler JE (2013). The emergence of a positive gerontology: from disengagement to social involvement. The Gerontologist.

[CR23] Löfqvist C, Nygren C, Iwarsson S, Szeman Z (2005). Assistive devices among very old persons in five European countries. Scandinavian Journal of Occupational Therapy.

[CR24] Marmot M, Allen J, Bell R, Bloomer E, Goldblatt P (2012). WHO European review of social determinants of health and health divide. The Lancet.

[CR25] Oswald F (2005). Neuropsychological aging inventory (NAI): manual.

[CR26] Oswald F, Mollenkopf H, Wahl HW (1999). Questionnaire on the meaning of home.

[CR27] Oswald F, Schilling O, Wahl HW, Fänge A, Sixsmith J, Iwarsson S (2006). Homeward bound: introducing a four-domain model for perceived housing in very old age. Journal of Environmental Psychology.

[CR28] Oswald F, Wahl HW, Schilling O, Iwarsson S (2007). Housing-related control beliefs and independence in activities of daily living in very old age. Scandinavian Journal of Occupational Therapy.

[CR29] Oswald F, Wahl HW, Schilling O, Nygren C, Fange A, Sixsmith A, Sixsmith J, Szeman Z, Tomsone S, Iwarsson S (2007). Relationships between housing and healthy aging in very old age. The Gerontologist.

[CR30] Ploubidis GB, Dale C, Grundy E (2012). Later life health in Europe: how important are country level influences?. European Journal of Ageing.

[CR31] Sonn U, Asberg KH (1991). Assessment of activities of daily living in the elderly. A study of a population of 76-year-olds in Gothenburg, Sweden. Scandinavien Journal of Rehabilitation Medicine.

[CR32] Sullivan M, Karlsson J, Bengtsson C, Furunes B, Lapidus L, Lissner L (1993). The Goteborg quality of life instrument -a psychometric evaluation of assessments of symptoms and well-being among women in a general population. Scandinavian Journal of Primary Health Care.

[CR33] Sullivan M, Karlsson J, Ware JE (1995). The Swedish SF-36 health survey--I. Evaluation of data quality, scaling assumptions, reliability and construct validity across general populations in Sweden. Social Science Medicine.

[CR34] Tomsone S, Horstmann V, Oswald F, Iwarsson S (2013). Aspects of housing and perceived health among ADL independent and ADL dependent groups of older people in three national samples. Aging and Clinical Experimental Research.

